# Supplementary figures and images for: CBP/p300 activation promotes axon growth, sprouting, and synaptic plasticity in chronic experimental spinal cord injury with severe disability
Source: PLoS Biol. 2022 Sep 20;20(9):e3001310. doi: 10.1371/journal.pbio.3001310 (PMC9488786; doi:10.1371/journal.pbio.3001310)

**A**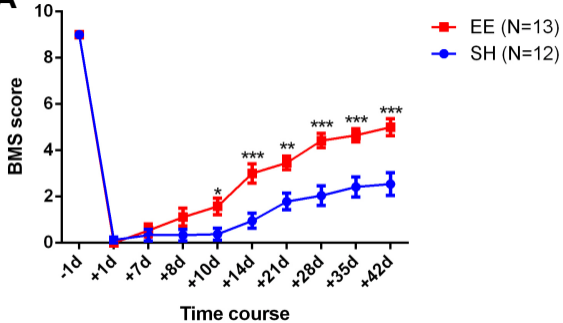**B**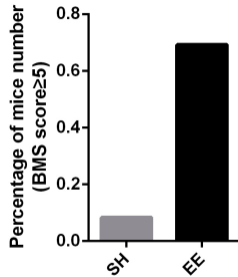**C**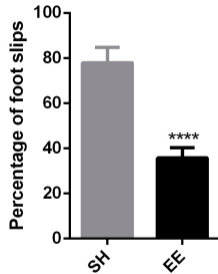

Supplement: S1 Fig — (A) Twelve weeks old mice were housed in SH or EE 1 week after a spinal cord transection. (B and C) Animals in SH remained impaired unable to step until day 42 after injury as shown by BMS (B) and Gridwalk (C). EE significantly enhanced locomotion (mean ± SEM, two-way ANOVA, Fisher LSD post hoc ** P < 0.01; *** P < 0.005; **** P < 0.001). The data can be found in S1 Data. BMS, Basso Mouse Scale; EE, enriched environment; SH, standard housing. (PDF) [file pbio.3001310.s001.pdf]

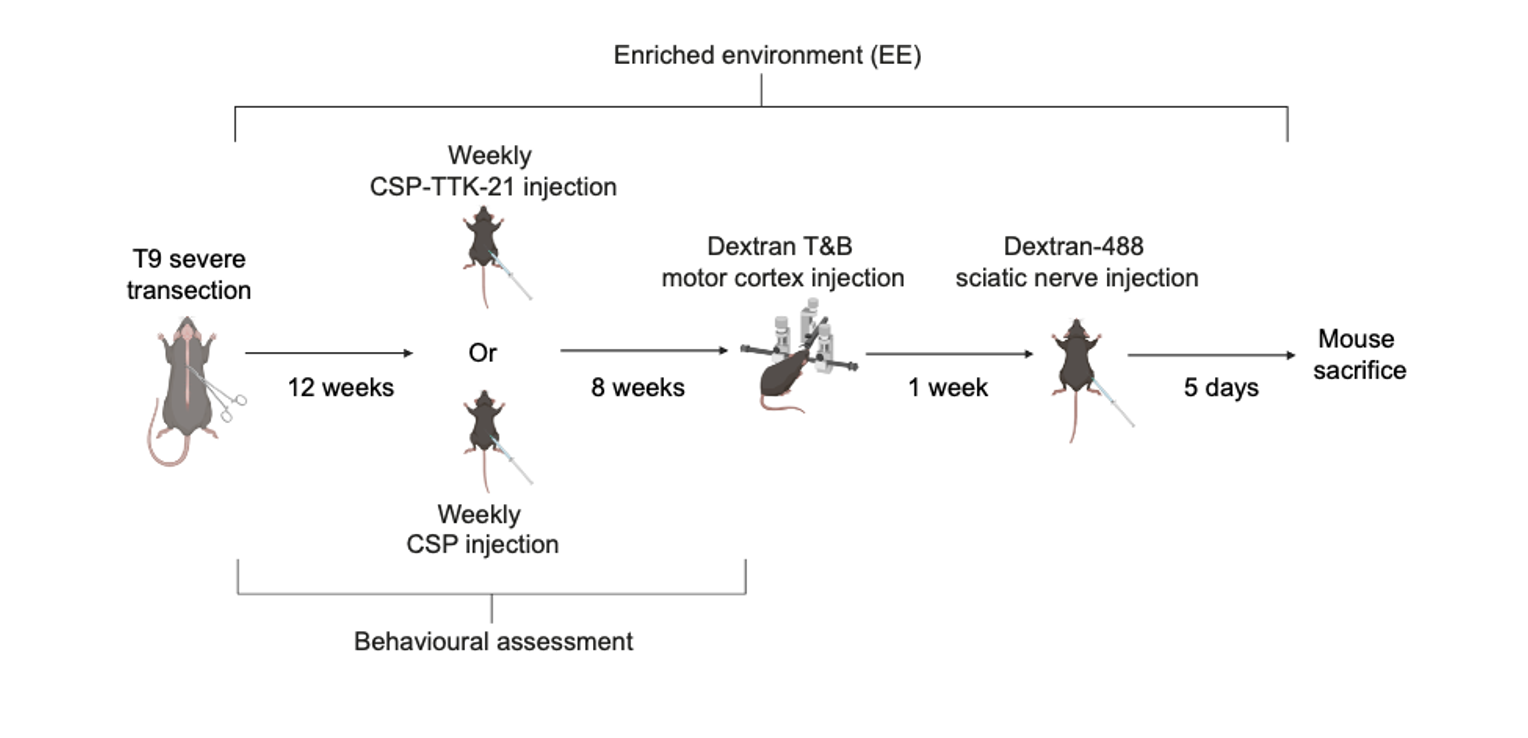

Supplement: S2 Fig — (PNG) [file pbio.3001310.s002.png]

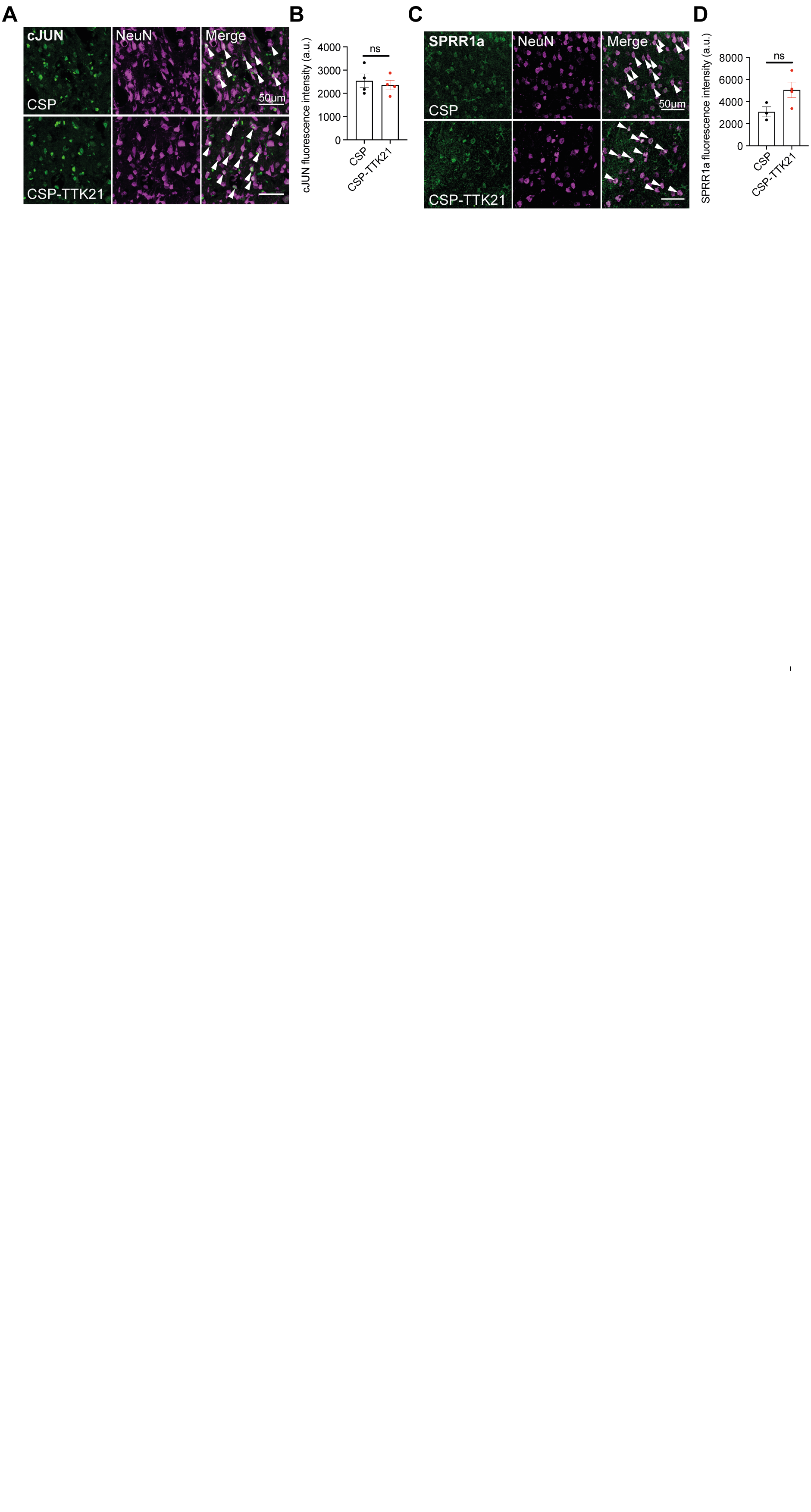

Supplement: S3 Fig — (A) Representative micrographs of cJUN immunostaining (green, white arrows) in layer 5 cortical neurons from CSP or CSP-TTK21-treated mice. (B) Quantification of cJUN immunostaining in layer 5 cortical neurons (CSP: 2,539.0 ± 295.4; CSP-TTK21: 2,355.0 ± 206.7, p = 0.63, TOST: t(0.1) = 0.1, p = 0.5 given equivalence bounds of −153.0 and 153.0 on a raw scale and an alpha of 0.05, n = 4). (C) Representative micrographs of SPRR1a immunostaining (green, white arrows) in layer 5 cortical neurons. (D) Quantification of SPRR1a immunostaining in layer 5 cortical neurons in CSP or CSP-TTK21 (CSP: 3,940.0 ± 491.6, n = 3; CSP-TTK21: 3,636.0 ± 350.3, n = 4; p = 0.08, TOST: t(4.8) = −1.9, p = 0.94 given equivalence bounds of −344.4 and 344.4 on a raw scale and an alpha of 0.05). Mean ± SEM; unpaired two-tailed Student t test or Welch t test. n = biologically independent animals. The data can be found in S1 Data. CSP, carbon nanosphere; TOST, two one-sided tests. (PNG) [file pbio.3001310.s003.png]

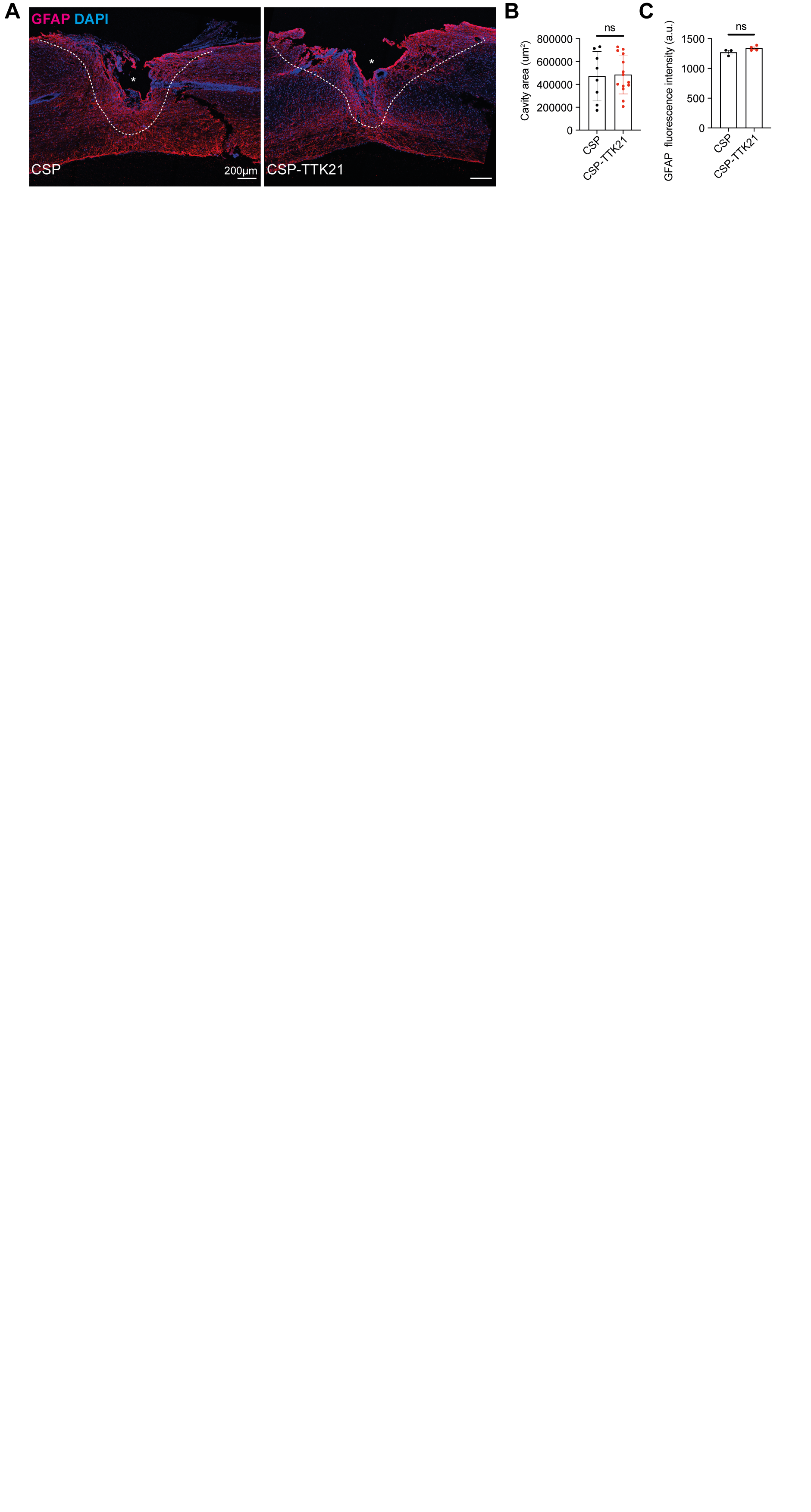

Supplement: S4 Fig — (A) Representative micrographs of GFAP intensity (red) around the SCI site (white asterisks) and cavity size (white dotted line) in CSP or CSP-TTK21 mice. (B) Quantification of cavity size in CSP or CSP-TTK21-treated mice (CSP: 471,574.0 ± 76,631.0, n = 8; CSP-TTK21: 486,466.0 ± 45,491.0, n = 14; p = 0.87; TOST: t(20.0) = 0.5, p = 0.31 given equivalence bounds of −56,344.2 and 56,344.2 on a raw scale and an alpha of 0.05). (C) Quantification of GFAP intensity in CSP or CSP-TTK21-treated mice (CSP: 1,272.0 ± 31.3, n = 3; CSP-TTK21: 1,339.0 ± 20.8, n = 4, p = 0.12, TOST: t(3.7) = −1.4, p = 0.88 given equivalence bounds of −14.5 and 14.5 on a raw scale and an alpha of 0.05). Mean ± SEM; unpaired two-tailed Student t test or Welch t test. n = biologically independent animals. The data can be found in S1 Data. CSP, carbon nanosphere; SCI, spinal cord injury; TOST, two one-sided tests. (PNG) [file pbio.3001310.s004.png]

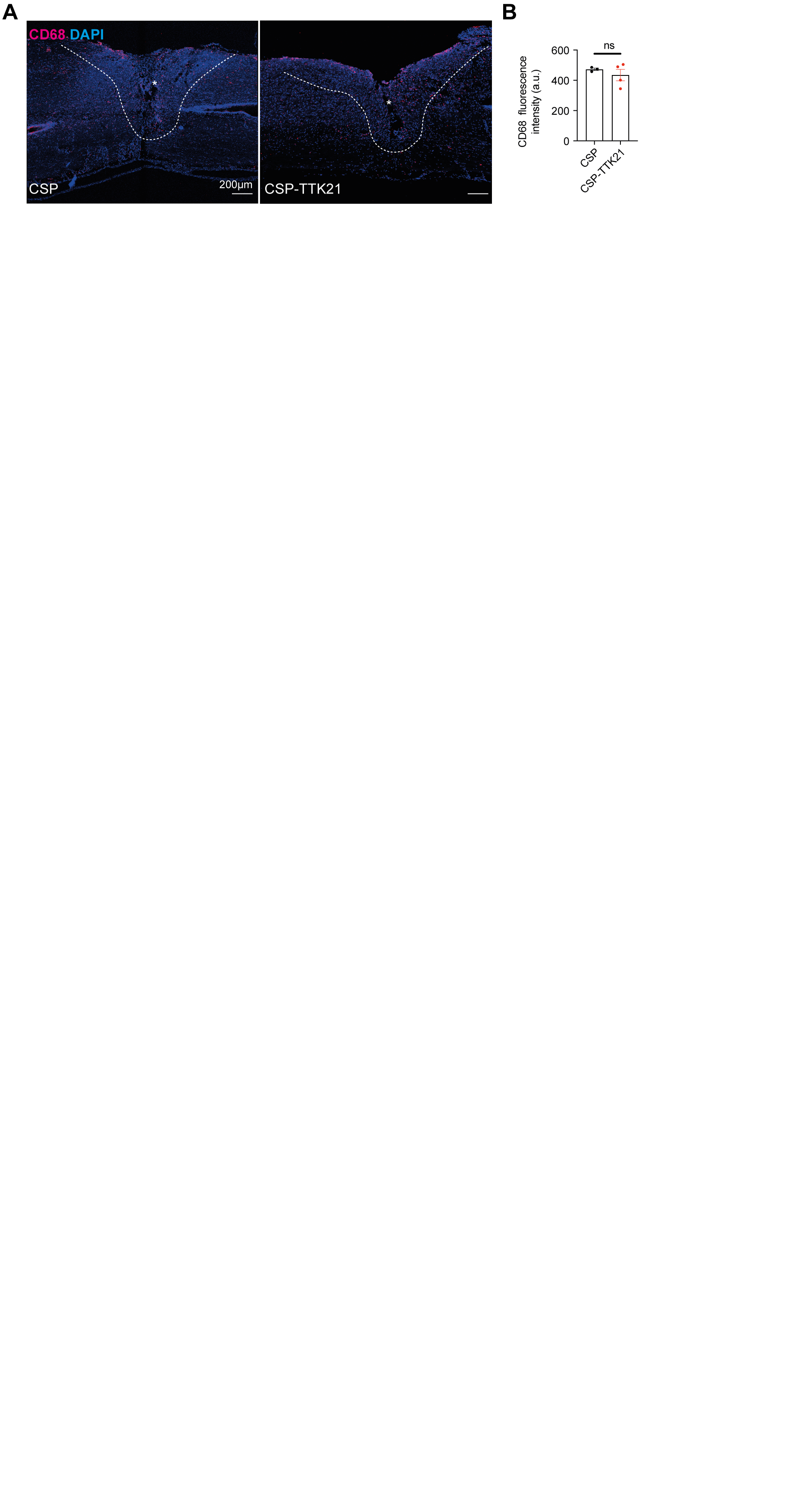

Supplement: S5 Fig — (A) Representative micrographs of CD68 immunofluorescence (red) and DAPI (blue) around the SCI site (white asterisks) in CSP or CSP-TTK21-treated mice. Lesion site (white dotted line). (B) Quantification of CD68 intensity in CSP or CSP-TTK21-treated mice (CSP: 472.0 ± 8.1, n = 3 CSP-TTK21: 434.5 ± 37.8 n = 4, p = 0.44, TOST: t(3.3) = 0.5, p = 0.69 given equivalence bounds of −16.3 and 16.3 on a raw scale and an alpha of 0.05). Mean ± SEM; unpaired two-tailed Student t test. n = biologically independent animals. The data can be found in S1 Data. CSP, carbon nanosphere; SCI, spinal cord injury; TOST, two one-sided tests. (PNG) [file pbio.3001310.s005.png]
