# Supplementary material for: CBP/p300 activation promotes axon growth, sprouting, and synaptic plasticity in chronic experimental spinal cord injury with severe disability
Source: PLoS Biol. 2022 Sep 20;20(9):e3001310. doi: 10.1371/journal.pbio.3001310 (PMC9488786; doi:10.1371/journal.pbio.3001310)
Supplement: Attachment — Submitted filename: Plos Biol response to reviewers 18-8-21 .docx [file pbio.3001310.s007.docx]

Dear Dr. Gasque,

Thanks very much for your editorial input and handling of our manuscript so far.

Please find here below our point-by-point rebuttal to your comments and to the reviewers’ points.

1) Regarding reviewer 3's recommendation to use rats instead of mice, we do not think that it is necessary that you switch your model system as recommended. However, you should be reserved in your discussion of the true clinical impact of your work.

A sentence in the manuscript addresses this point (page 7)

2)	We agree with reviewer 3 that four groups should be tested for this study, even though previous studies have completed two of the groups.

We have clarified our position in the rebuttal here below, including the offer to perform staged experimental groups -adding 2 groups in case of positive outcomes with the CBP/p300 TTK21 activator, based upon experimental and regulatory (Home Office) reasoning.

3)	We also think that negative results may be difficult to interpret. Thus, we would recommend that you are reserved in the conclusions you draw from the study given that hierarchy of synergy is not thoroughly tested. You may be able to address this with a clearer rationale of the treatment paradigm, as was requested by all the reviewers, but see point (5) below.

We fully agree that this needed improvement and clarification, also affecting point 2 here above. This has been clarified in the rebuttal here below and in the text (pages 5-7).

4)	We agree with the concerns of the reviewers regarding positive controls and sample size, which go to the heart of the criteria for accepting a Registered Research Articles Stage 1. It will be important for you to thoroughly address these points (among the many others). We have addressed these and the other points

5)	We would also like to see a more comprehensive specification of the proposed frequentist equivalence tests and/or Bayesian hypothesis tests (including, especially, the choice of prior for the Bayesian tests and justification of that prior) and the chosen parameters for the frequentist tests. You also need to be clear which tests, Bayesian or frequentist equivalence, will determine the interpretation in the event of negative results, as it is not guaranteed that they will produce equivalent strength of evidence.

While we have prior data in acute spinal cord injury to justify a Bayesian approach, the experimental design is not the same, although similar, and one of the major limitations that Bayesian methods face is that the prior distributions can potentially bias our statistical results.

Therefore, our priority would be to adopt frequentist equivalence test. There are 5 parameters: sample size(n), difference between the groups (delta), standard deviation (sigma), type I error (alpha/significance), and type II error (power). We can use estimates for 4 of these parameters to calculate the 5^th^ parameter – which we have done to achieve our required sample size to achieve a certain power and significance. We have added a sentence in the manuscript with regards to this.

Reviewer #1:
There are only a few minor suggestions which could be addressed to further clarify the study.
-The authors had previously proposed that EE-induction of lasting increase in regeneration potential is mediated by a Cbp-dependent increase in histone acetylation and increase in gene expression, including pathways involved in neuronal activity, axonal projection and cytoskeleton remodeling. While EE likely affects multiple pathways and mechanisms that might act positively to improve SCI outcomes, the authors indicated that Cbp-dependent mechanisms likely mediate EE effects. As such, it is less clear why a strong synergy is expected when EE and the drug are combined.

**This reviewer is absolutely correct to raise this point that deserves further clarification. Indeed, combined proteomics and transcriptomics studies that we performed for a previous publication indicate that the potential benefit of EE in terms of pro-plasticity and regenerative signalling goes well beyond CBP pathways as reported in Hutson et al., (Science Trans Med, 2019). They include modulation of mitochondrial metabolism, calcium signalling, ion channels, axonal transport and release of extracellular signalling vesicles among others. This information has also been added to the manuscript** (pages 5-7)**.**

-The sex of mice that will be used in this study is not mentioned.

**Female animals will be used to better allow housing of several animals together given their much lower propensity to fight- this way we avoid issues of aggression/fighting that can lead to injury/death thus forcing us to separate mice, which would contradict the whole point of EE as providing an enriched social and physical environment. The use of female mice has been added in the manuscript (page 11).**

-Expected timeline for the completion of the study was not included but would be helpful.

**The timeline is expected to be of approximately 6 months**

-The author might want to consider using Minimum Information about a Spinal Cord Injury Experiment (PMID: 24870067).

**Thanks for this. We will make sure to comply with this upon completion of the experiments and reporting of the results**

Reviewer #2:
There are some conceptual and technical issues that arise. I emphasize again the potential importance and novelty of this study; the comments below are offered in the spirit of helpful refinement or simply providing the authors a chance to help me better understand the rationale for some details.

1.	The largest conceptual question: what is the rationale for supplying enriched environments one week after injury, but delayed CBP stimulation? And the closely related question: if the model is that EE is acting through a CBP mechanism, what is the rationale for providing EE to all animals and then additional CBP to half? The concern is that if EE already maximally engages the CBP-dependent pathway, there will be no added benefit to the pharmacological treatment. In general there is potential confusion here about the relationship between the mechanisms of the two treatments, how that relationship affects the rationale of their dual use, and how that relationship affects the selection of their timing.

**Thanks for this important point of discussion. It gives us a chance to better explain our rationale and the experimental design.** **EE does not represent a specific form of focused rehabilitation, but rather a more physiological setting compared to (standard housing) SH that better reflects the human condition where patients are encouraged to engage in physical activities early on after a spinal cord injury. This explains the rationale for having animals in EE as baseline as opposed to having them in SH, which in reality represents an impoverished artificial environmental setting. Indeed, our study will test the effect of the CBP activator on this more “physiological” background in chronic severe SCI.**

**We have modified the text to make this clearer and more compelling (page 5-7).**

**This reviewer correctly raises the point of the EE and CBP pathways that partially overlap. While this is true, combined proteomics and transcriptomics studies that we performed for a recent publication, indicate the potential benefit of EE in terms of triggering physiological mechanisms that might promote pro-plasticity and regenerative signalling that go well beyond CBP pathways as reported in Hutson et al., (Science Trans Med, 2019). They include modulation of mitochondrial metabolism, calcium signalling, ion channels, axonal transport and release of extracellular signalling vesicles among others. This information has also been added to the manuscript (page 5-7).**

2.	Why is the CST being traced and analyzed, as opposed to the Gi as previously? The Gi is likely more relevant to locomotion, and there is already data to support some effect. The jump to CST is more likely to yield negative results and less likely to be related to the behaviors in question.

**Thanks for this, we agree with the reasoning. However, please consider the reasons that led us to make our choice:**

1. **Reticulospinal tracing is extremely challenging in the mouse**
2. **We will investigate 5-HT axonal tracts that partially functionally overlap with the reticulospinal**
3. **The choice of the CST lies on the fact that these tracts are important for motor function in humans, and we would like to challenge their growth/plasticity potential with our intervention to possibly build upon this study for future more CST targeted models**

**Lastly, we will attempt reticulo-spinal tracing and if successful this can be added to the design.**

3.	The study seems to lack positive controls. Given the extreme challenge of promoting axon growth in the chronic injury condition, negative results are quite possible. To plan for this, it seems important to have positive controls in place, especially for the histone and gene expression measurements.

**Thanks for this valid point. Indeed, we will measure histone acetylation after the delivery of the TTK21 as positive control since this has been shown to promote histone acetylation previously by activating CBP/p300 activity.**

4.	A smaller technical question - why BDA for tracing CST axons? Viral tracing methods, used previously by this group, would seem to offer more sensitive detection of fine collaterals in the spinal grey matter. But perhaps this is not the case, or there may be other technical considerations that led to the selection of BDA?

**Our tracing is technically speaking Dextran, Tetramethylrhodamine and biotin, 10,000 MW, Lysine Fixable (mini-Ruby) and it is of high quality giving excellent tracing in our hands** (<https://www.thermofisher.com/order/catalog/product/D3312#/D3312>)

**-we have specified this in the text as opposed to the more general BDA denomination. However, we will follow the suggestion of this and of reviewer 3 and we will compare these alternative tracing methods that will be used if they prove to be superior to the above tracer.**

Reviewer #3, Mark H. Tuszynski: This is an interesting proposal to study a combination of a slow-release nanoparticle to activate CREB-binding protein (CSP-TTK21) and environmental enrichment (EE) in mice after spinal cord injury (SCI).  The work is proposed by a careful group that publishes high quality, well-documented studies in high impact journals.
The Di Giovanni group has previously published that EE enhances sensory axonal growth and motor behavioral recovery after SCI: EE increases H4K8ac protein levels and phosphorylation of CREB (Hutson TH et al, Sci Transl Med, 2019), as shown in Supporting Fig 1.
I have one major comment regarding the experimental design, several moderate comments, and some minor comments.
Major: Experimental groups - there will only be two experimental groups even though 2 therapies will be applied.  I believe that for the emerging data from this study to be clearest and of the highest quality, there should be 4 groups:  1) Untreated control lesioned.  2) Treatment with EE.  3) Treatment with TTK21.  4) EE + TTK21.  The authors may consider their previous report regarding the benefit of EE alone to be sufficiently compelling that they need not show the value of this alone, but I do not agree.  This is a different study and there may be different effect sizes of EE, and synergies between EE and TTK21 that are only evident by examining adequate controls for each treatment.

Moderate: The authors reference "chronic" injury at several points of the paper, but EE is initiated one week after the injury in the proposed studies, not late, and a 3 month post-injury time point is not in any case necessarily "chronic". The authors might consider relying less heavily on this term.  Perhaps more importantly, the authors might consider starting BOTH therapies 3 months after injury, or at least starting EE one month after injury: the average time in the U.S. that most patients enter rehabilitation is one month after injury, and few begin rehab one week after injury.  But if the authors truly wish to study a late time point after injury when immediate post-injury events have subsided, initiating both therapies at 3 months would be better, in my opinion.

**Thanks for raising these very interesting points that make for a very interesting discussion. I will address the 2 above points together since their rationale is closely linked.**

**Timelines in rodents are much tighter than in humans as is their life span: much shorter; more specifically, both axonal growth during development and during repair processes are much faster in rodents than humans as are the wound healing responses. Therefore, one week in a mouse cannot be considered as one week in a human. One week in rodents is typically comparable to several weeks in humans- although of course there is no perfect equation to measure this with absolute rigor. It is important to note that rodents age quickly and waiting much longer than in our experimental design comes with the risk of having aging-dependent limitations on repair overlapping with the chronicity of the lesion.**

**Here we would like to clarify our point of view on EE and we apologise for not having made it more clear in the text.**

**As previously mentioned, EE does not represent a specific form of focused rehabilitation, but rather a more physiological setting, compared to (standard housing) SH, that better reflects the human condition where patients are encouraged to engage in physical activities early on after a spinal cord injury. This explains the rationale for having animals in EE one week after injury as baseline physical and environmental more physiological conditions, as opposed to standard housing, which in reality represents an impoverished artificial environmental. Indeed, our study will test the effect of the CBP activator on this more “physiological” background in chronic severe SCI, where we believe we can additionally take advantage of potential synergistic effects of EE and CBP activation.**

**In order to respect the rules of reduction (N of animals and experimental groups) and refinement (EE vs SH) according to animal experimentation guidelines and as recommended and permitted by the UK Home Office for protocols of this severity, we have been allowed to complete a first study where we can assess whether CBP activation on an EE background will improve neurological performance and/or neuroanatomical outcomes (one independent variable) as described. If that will be the case, we will be allowed to complete the study by performing the 2 additional groups, as requested by this reviewer.**

**We have modified the text to make this reasoning clearer and more compelling (pages 5-7).**

Sampling Plan:  The authors will study only 6 animals per group based on a power calculation that assumes a 200% increase in axon growth between the control and treatment group.  This is very optimistic!  I strongly suggest that the authors consider increasing the N per group to 12.

The functional studies also propose an effect size of 100%, which once again is quite optimistic (notwithstanding the preliminary data in Supp Fig 1). Again, I would increase group size to 12.

**The group size was calculated using power calculations with differences based upon the published study in acute SCI (Hutson et al, 2019). However, we agree that the difference in axon growth obtained in acute might not be representative of a more chronic model, and that it can therefore be considered optimistic. If we reduce the predicted difference by 50%, with a power 90% as previously set, we will be required to increase the N to 8. However, in the case of behavioural studies, given the higher variability, we are happy to bring the group size to 12 here. We have modified the text accordingly (table and statistical section).**

The authors propose to use the mouse model. Why use this small animal species as opposed to the rat?  Data in rats may be more clinically relevant, and the authors state that they wish to design a clinically relevant experiment.

**We appreciate this very important point. In fact, we have carefully considered the choice of the model at this point in time, and we have explained it in our manuscript. This reviewer might have missed the following section. Pasted here for his convenience:**

“Since no data is available on TTK21 in chronic SCI at this stage, a severe transection model in mice has been chosen versus a contusion in rats because (i) it will allow a more accurate anatomical definition of axonal regeneration and sprouting; (ii) it has much less variability allowing for a more reliable statistical assessment keeping the number of animals relatively limited and (iii) it will need a fraction of the drug required (in mice vs rats) that is produced by our long-standing collaborator Professor Tapas Kundu (Centre for Drug Discovery, India). Currently the availability of the compound does not allow to initiate studies in rats in the short term, however these studies will surely prompt further investigation in severe chronic contusion in rats.”

Minor:
The authors state that they will use a "severe" transection model of SCI, but a dorsal over hemisection is not that severe.  Mouse with this injury spontaneously recover to a BMS score of nearly 3, are show a gradual improvement in function over 42 days, although the authors state that this model causes "permanent" deficits. These lesions spare the ventral motor tracts of the spinal cord (esp reticulospinal) which can support several motor behaviors. These statements do not entirely correspond to the presented data in Supporting Figure 1, and some clarification is needed both with regard to exactly what the lesion is, and why it is considered permanent.

**In recent experiments, we have performed a deep lesion that transects the spinal cord dorsal to ventral sparing only a small portion of the ventral spinal cord, which leaves the animals with a severe and permanent neurological impairment- no improvement from baseline. We have clarified this in the manuscript (methods, page 12).**

The authors propose to use BDA as a tracer of the corticospinal system. Generally, our experience has been that newer fluorescent tracers such as TdTomato or membrane-trafficked tracers expressing fluorophores provide superior corticospinal axon visualization. The authors might consider using these tracers.

**Our tracing is technically speaking Dextran, Tetramethylrhodamine and biotin, 10,000 MW, Lysine Fixable (mini-Ruby) and it is of high quality giving excellent tracing in our hands** (<https://www.thermofisher.com/order/catalog/product/D3312#/D3312>) (page 8).

**-we have specified this in the text as opposed to the more general BDA denomination. However, we will also follow the suggestion of this and of reviewer 3 and we will compare these alternative tracing methods that will be used if they prove to be superior to the above tracer.**

The authors propose to perform immunolabeling for a number of RAGs to gauge expression levels: this is some variability in these immunolabeling methods. Might it be possible to pursue a less extensive list of RAGs and study levels by, e.g., Western blot of quantitative in situ hybridization?
The authors inconsistently state that they will trace central projections of DRG axons with CTB in one part of the description, and Dextran red in another.

**While variability might be an issue, our lab has extensively performed IF for several RAGs and this is an advantage since it allows us comparison with previous datasets. In case we encounter excessive variability in signal intensity we can do WB as suggested although we will miss cell specificity in that case.**

**Apologies about this. Dextran 488 will be used; the text has been amended as needed (page 8).**

The authors provide supporting data in support of EE enhancing motor recovery. However, they do not provide anatomical data that supports their claim of axonal/synaptic reorganization.  According to the text, this should be included in supporting figure 1.

**Since the lesion we will be performing might be more severe than the one presented in the supporting data we will leave it as it is as proof of principle, however we will provide anatomical data when the new experiments have been completed**

For 5-HT immunolabelling, it is probably unnecessary to post-fix in glut, then perform antigen retrieval.  In our experience, anti-5-HT antibodies (from Immunostar) perform well with 4% pfa fix without antigen retrieval.

**Thanks for this useful suggestion. Actually, talking with the team I found out that some in the lab are indeed doing what this reviewer suggests already, the text has been amended as needed (page 13).**

Behavioral analysis:  Assessments will be performed on week 1 post-injury and weekly thereafter, and CSP-TTK21 injections will be performed on the same day. I suggest that the motor assessments be performed prior to the IP injections.

**Thank you, this makes sense, we will do.**

Interpretations of negative outcomes:  The authors very nicely propose several possible interpretations of negative outcomes (i.e., a lack of anatomical, behavioral or molecular differences from controls). But since this is a combinatorial treatment paradigm, there may in fact be many additional reasons that the approach could yield data. While all possibilities for a negative outcome are likely to be elucidated in this study, it remains worthy of study.

**Thanks for this.**

Overall, this is an interesting study that appears likely to yield useful and potentially important data.  I hope that the preceding suggestions are helpful.

Thanks for the consideration

Simone Di Giovanni
