# Supplementary material for: CBP/p300 activation promotes axon growth, sprouting, and synaptic plasticity in chronic experimental spinal cord injury with severe disability
Source: PLoS Biol. 2022 Sep 20;20(9):e3001310. doi: 10.1371/journal.pbio.3001310 (PMC9488786; doi:10.1371/journal.pbio.3001310)
Supplement: Attachment — Submitted filename: Mueller et al rebuttal Plos Biology revised 16-09-21.docx [file pbio.3001310.s008.docx]

Division of Brain Sciences

Imperial College London

Room E505

5^th^ Floor, Burlington Danes

Hammersmith Hospital Campus

Imperial College London

Du Cane Road, W12 0NN

Tel: +44 020 759 43178

[s.di-giovanni@imperial.ac.uk](https://exchange.imperial.ac.uk/owa/redir.aspx?C=Ab4gaWRkekCYKooeouNOMQCZKJ_gpNAISB8XdSK4TgWo7MKdEA3nCgNCT2AC6q0lv-qmHy__DyE.&URL=mailto%3as.di-giovanni%40imperial.ac.uk)

[www.imperial.ac.uk](http://www.imperial.ic.ac.uk)

Dr. Gabriel Gasque

Senior Editor

Plos Biology	Simone Di Giovanni

Professor of Restorative Neuroscience

London, 16-09-2021

**Reviewer 2.** One could quibble and point out that the rationale offered for combining EE and CBP stimulation somewhat missed the question. The authors have clarified that EE does not act solely through CBP; that is quite clear and was not the source of confusion. The question was the opposite - are we sure that CBP activation via CSP-TTK21 is offering a benefit beyond the stimulation of CBP already achieved through EE? In other words, is the pharmacological effect on CBP somehow stronger or broader than EE's effect on CBP? If not, the drug is simply duplicating an effect already present in EE animals. But on reflection, a stronger effect from the drug seems likely enough that the overall experiment is interesting and very worthwhile. I clarify this point only to sensitize the authors to the likelihood that other readers may wonder the same thing - not just whether EE is doing more than just activating CBP, which is now clarified in the revised manuscript, but also whether CSP-TTK21 is doing more to CBP than EE alone, which does not seem to be addressed. It would seem that both must be true to predict synergy.

CBP activation via CSP-TTK21 leads to a much more robust increase in histone acetylation and regenerative gene expression than EE alone by activating both CBP and p300 histone acetyltransferases.

        Finally, in response to questions about positive controls for histone and gene expression outcomes, the authors reiterated the intention to measure histone acetylation, pointing to prior findings that CSP-TTK21 treatment increased H4K8ac. Again, this was understood when the question was raised. This prior finding was obtained by acute delivery, not in the chronic state, so even in a narrow sense it is not clear that this readout qualifies as a positive control per se; it seems more of an open question, whether the histones of chronically injured neurons will be modified in the same way. More broadly, it leaves unanswered any question about positive controls for the RAGs to be measured. In reading responses to other reviewers, it appears that the intention is to compare the immunofluorescence obtained in these samples to other datasets obtained previously in the lab. In light of the outstanding and extensive track record of the lab, this is probably acceptable, although of
course it must be done cautiously.

Thank you for the above discussion
