# Supplementary material for: CBP/p300 activation promotes axon growth, sprouting, and synaptic plasticity in chronic experimental spinal cord injury with severe disability
Source: PLoS Biol. 2022 Sep 20;20(9):e3001310. doi: 10.1371/journal.pbio.3001310 (PMC9488786; doi:10.1371/journal.pbio.3001310)
Supplement: Attachment — Submitted filename: Mueller et al rebuttal Plos Biology full sub.docx [file pbio.3001310.s009.docx]

Division of Brain Sciences

Imperial College London

Room E505

5^th^ Floor, Burlington Danes

Hammersmith Hospital Campus

Imperial College London

Du Cane Road, W12 0NN

Tel: +44 020 759 43178

[s.di-giovanni@imperial.ac.uk](https://exchange.imperial.ac.uk/owa/redir.aspx?C=Ab4gaWRkekCYKooeouNOMQCZKJ_gpNAISB8XdSK4TgWo7MKdEA3nCgNCT2AC6q0lv-qmHy__DyE.&URL=mailto%3as.di-giovanni%40imperial.ac.uk)

[www.imperial.ac.uk](http://www.imperial.ic.ac.uk)

Simone Di Giovanni

Professor of Restorative Neuroscience

London, 03-06-2022

Dear Reviewers,

I am pleased to submit our fully executed manuscript now entitled **“CBP/p300 activation promotes axon growth, sprouting and synaptic plasticity in chronic severe experimental spinal cord injury”** for consideration at Plos Biology.

As you will remember this manuscript has been accepted as preregistered article last year before we experimentation.

I am excited to share our results that provide evidence that it is possible to promote axonal growth, sprouting and synaptic plasticity in a chronic and severe SCI. In fact, the pharmacological activation of CBP/p300 promotes regenerative signalling, axonal growth, sprouting and synaptic plasticity of both sensory and motor axons. However, this is not sufficient to promote sensorimotor recovery. We report these findings, and we also discuss the limitations, the opportunities and the research directions that this work suggests.

Please find the fully executed revised manuscript (with track changes and a clean one).

New sections include results, discussion and figure legends (not tracked since they are new).

The title has been modified to better reflect the experimental findings. We have followed what planned, no modification to the planned experiments has been done.

Looking forward to the final review of our work**.**

Thank you

With my best wishes,

Simone Di Giovanni
