# Supplementary material for: CBP/p300 activation promotes axon growth, sprouting, and synaptic plasticity in chronic experimental spinal cord injury with severe disability
Source: PLoS Biol. 2022 Sep 20;20(9):e3001310. doi: 10.1371/journal.pbio.3001310 (PMC9488786; doi:10.1371/journal.pbio.3001310)
Supplement: Attachment — Submitted filename: Mueller et al Rebuttal Plos Biol minor rev 09-08-22.docx [file pbio.3001310.s011.docx]

Division of Brain Sciences

Imperial College London

Room E505

5^th^ Floor, Burlington Danes

Hammersmith Hospital Campus

Imperial College London

Du Cane Road, W12 0NN

Tel: +44 020 759 43178

[s.di-giovanni@imperial.ac.uk](https://exchange.imperial.ac.uk/owa/redir.aspx?C=Ab4gaWRkekCYKooeouNOMQCZKJ_gpNAISB8XdSK4TgWo7MKdEA3nCgNCT2AC6q0lv-qmHy__DyE.&URL=mailto%3as.di-giovanni%40imperial.ac.uk)

[www.imperial.ac.uk](http://www.imperial.ic.ac.uk)

Dr. Kris Dickson

Senior Editor

Plos Biology	Simone Di Giovanni

Professor of Restorative Neuroscience

London, 09-08-2022

Dear Dr. Dickson,

I am pleased to submit the point by point rebuttal with regards to our revised manuscript entitled **“CBP/p300 activation promotes axon growth, sprouting and synaptic plasticity in chronic experimental spinal cord injury with severe disability”** for publication in Plos Biology.

Thank you for your consideration

With my best wishes,

Simone Di Giovanni

Reviewer #1: In this study, the authors investigated whether delayed delivery of CBP/p300 activator TTK21 in adult mice after severe transection SCI in combination with enhanced environment (EE) housing promotes histone acetylation, axonal and synaptic plasticity and behavioral recovery.

Sprouting and regeneration of the dorsal columns and CSTs were analyzed with the retrograde and anterograde axonal tracers, respectively. Axonal dieback as well as the

number of fibres past the lesion site were normalized to the number of labelled fibres prior to

the lesion. The authors measured sprouting of serotoninergic raphe-spinal motor tracts with 5-HT immunohistochemistry. To assess whether the CSP-TTK21 treatment enhances synaptic

plasticity, they measured the number of inhibitory vGat or excitatory vGlut1 synaptic terminals

in proximity of neuronal targets such as interneurons in the dorsal horns and motoneurons in

the ventral horns of the spinal cord (ChAT or NeuN immunostaining). Histone acetylation as read out of CBP/p300 activation was evaluated in layer V neurons, raphe nuclei and DRG neurons by immunofluorescence. The expression of several regeneration associated factors including ATF3, JUN, GAP43, SPRR1a, KLF7, pERK, and pSTAT3 was studied by immunofluorescence in sensory and motor neurons. Locomotion, coordination and sensorimotor integration were measured by performing open field assessment with the BMS and the gridwalk tests. Von Frey test for mechanoception and mechanical allodynia as well as Hargreaves test for thermoception and thermal hyperalgesia were used to specifically assess the function of the ascending sensory tracts. The authors conclude that TKK21 treatment promotes histone acetylation in both DRG and cortical neurons, increases expression of some RAGs in DRGs, prevents axonal dieback, promotes axonal sprouting, particularly for 5-HT axons. However, recovery of function was not observed in these TTK21-treated mice.

This study was set out to address an important question of whether previously identified regenerative treatment as shown in acute SCI models can promote plasticity and recovery in chronically injured animals. Importantly, the authors reason that EE does not represent a specific form of focused rehabilitation, but rather a more physiological setting compared to (standard housing) SH that better reflects the human condition where patients are encouraged to engage in physical activities after a spinal cord injury. There were several suggestions raised by the reviewers in the first round of review, most of which were decided not to be taken in the final study. These suggestions include inclusion of additional control groups, performing EE treatment months after SCI (i.e. at the time of TTK21 treatment) to reflect a delayed treatment paradigm, and choice of anterograde tracing method. However, it can be viewed that the authors present reasonable justification at least for the latter two recommendations. From the study, the authors conclude that although TTK21 with EE promotes histone acetylation and expression of select RAGs in sensory neurons, this treatment paradigm alone is insufficient to promote recovery of functions. The authors discuss that the chronically injured environment of CNS and spinal cord might need further modifications to successfully induce functional recovery. The overall conclusion of the findings is an important addition to the SCI field and highlight the clear challenges for treating chronic SCI patients. The techniques used in this study are standard in the field and the authors have carried out carefully designed experiments to answer specific questions.

Minor points:

Figure 2A, there is a duplicate of NeuN image for the TTK21 group, and the ATF3 single channel image for the TTK21 group is missing.

**Thank you for spotting this, the figure has now been revised accordingly.**

Figure 3A-D, it is unclear if the rostral axons represent those labeled axons that have regenerated past the lesion site (i.e. distal to the lesion). If so, it is not easily seen these labelled axons in the representative images provided in Figure 3A and 3C.

**We have now provided revised images showing axons rostrally to the border of the lesion**

The authors should consider citing these papers which seem relevant to the idea tested (i.e. challenges and possibilities in treating chronic SCI) and to the discussion of the findings. PMID: 26134657, PMID: 11717367, PMID: 33975016

**PMID: 26134657 and PMID: 33975016 have been added as directly relevant to the discussion, thank you**

Reviewer #2: The introduction, rationale and stated hypotheses are the same as the approved Stage 1 Protocol submission and experiments were executed and analyzed as planned.

I do have some questions about the presentation and conclusions presented in the abstract and discussion.

1. Overall there may be some tendency to advertise positive results but leave it to the reader to dig out the limitations and the effect size (which is quite small). The abstract in particular should give a more balanced and quantitative view of the data. The fact that RAGs were activated in DRG but not CST neurons is a significant finding. The fact that DRG and CST axon sprouting averaged less than 500 micros from the PROXIMAL edge of the lesion, and apparently didn't extend into tissue beyond the lesion, is certainly relevant information and informs the lack of behavioral effect. Bottom line, in my view the abstract should provide information about differences between the responses by different cell types and quantitative reference to the effect size of growth.

**These are fair points. We have revised the abstract to be more balanced and specific, however, we prefer to leave the description of detailed quantitative data to the results sections, which is more appropriate than in the abstract in our view.**

2. The injury is not sufficiently described, specifically the distinction between this injury, which is described as "severe" and a prior injury. This is important, because the difference in injury is offered as an explanation for the apparent reduction in DRG growth response in the prior acute study and the current chronic study.

From the present manuscript:

"A laminectomy at vertebra T9 was performed to expose spinal level T9 and a deep dorsal transection past the central canal leaving using micro-scissors (Fine Science Tools)."

From the prior manuscript (Joshi et al. 2015).

"A laminectomy at vertebra T9 was performed to expose spinal level T12 and a dorsal hemisection until the central canal was then performed using micro-scissors (Fine Science Tools)."

A more rigorous description of how depth was monitored, and how the depth differed between the two studies, is needed to support the claim that it can explain the reduced growth present here.

**We have detailed this now. Briefly, the previous study had a depth of about 0.5 mm, which was described as “ a dorsal hemisection  until the central canal”.**

**The current dorsal transection past the central canal is an average of 1.1 mm deep as measured after histological analysis.**

3. There seems to be a claim that TTK21 has different efficacy in sensory and motor neurons.

"It is however important that TTK21 increases the growth and regenerative gene expression ability of both sensory and motor neurons, albeit with differential potency and efficacy."

Is this referring to a difference in the response of CST and DRG in the present data? I don't see that in the present data or a statistical test to support that claim.

**This refers to the differential effect upon RAGs between sensory and motor neurons and sprouting of motor axons such as the 5-HT fibers. Now rephrased to make it clearer**

4. "These findings are in line with previous work in subacute SCI where these classical RAGs were not activated in the corticospinal as opposed to DRG neurons[27]. "

The reference is Dr. Tuszynski's claim that CST neurons temporarily revert to an embryonic state, and doesn't seem to support the claim being made for differential gene activation in DRG versus CST neurons.

**What we meant here is that while CST neurons temporarily revert to an embryonic state, they do not re-express classical RAGs a shown here. This has now been reworded.**

Reviewer #3: Muller and colleagues report their followup study regarding CBP/p300 activation in a model of delayed SCI (T9 partial (?) transection 1and treatment 12 weeks after injury).

Abstract: The authors state: "The interruption of spinal circuitry following spinal cord injury disrupts neural activity AND IS FOLLOWED BY A FAILURE TO MOUNT AN EFFECTIVE REGENERATIVE GENE EXPRESSION RESPONSE resulting in permanent neurological disability." I believe that the statement in CAPS is not accurate. We reported in Nature 2020 that SCI DOES result in mounting regenerative gene expression, but the absence of a permissive milieu in the lesion site results in regenerative failure (Poplawski 2020; 581:77). I suggest that the authors simply state AND IS FOLLOWED BY A FAILURE TO MOUNT AN EFFECTIVE REGENERATIVE RESPONSE.

**Good points, modified as suggested**

Related to the comment above, the authors may wish to reframe their conceptual context. We found as stated above that the regenerative state after SCI lasts for two weeks only. In a chronic state of injury (12-22 weeks after injury), this "primed" state for regeneration has closed. Thus, the authors may, by delivery of CSP-TTK, re-open this regenerative window. This concept is important from my perspective because we need to identify means of re-opening the regenerative state.

**Agreed, added to discussion**

Experimental Design: This section states that anatomical transection in a mouse allows more accurate anatomical definition of regeneration and sprouting than contusion in rat. Please simply state that anatomical transection allows more accurate anatomical assessment of regeneration and sprouting, since the choice of mice or rats has no bearing on transection vs. contusion. Also the authors suggest that T9 transection may spare some axons. This is not the case is the lesions are accurately performed. The lesion images shown in the figures appear to spare a substantial amount of spinal cord tissue. I think this point needs clarification: either it is advisable to omit reference to "severe" SCI, or to clarify why the images that show a substantial amount of sparing in Fig 3 are "severe".

**These lesions lead to severe functional impairment as seen by the behavioural tests- this has been rephrased as necessary to avoid misinterpretations**

Regarding the finding that TTK increased axonal growth but not functional recovery: Yes, TTK significantly increased axon growth, but the effect size was not very large. One might think that this is the reason that function did not recover. Perhaps this should be stated simply and clearly. If one goes from no regenerating axons to a few, this may not be likely to exert much of a benefit on function.

**Agreed, stated now in the discussion**

Hypothesize is misspelled in the paper ("Hypothesise").

**That was British English spelling, happy to convert to American English**

I greatly appreciate that the authors are reporting the fact that these experimental manipulations do not improve functional outcomes. Too often in this field, these negative findings are omitted from a paper. This complete reporting enhances the qualify and credibility of the work.

**Our pleasure**
